# When physicians become patients: A podcast series for learning from patient experiences

**DOI:** 10.1016/j.pecinn.2025.100423

**Published:** 2025-08-22

**Authors:** M.A. van Helvoort, K.J.A. van Dijsseldonk, B. Post

**Affiliations:** a Department of Psychiatry, Radboud university medical center, Nijmegen, the Netherlands; b Department of Neurology, Radboud university medical center, Nijmegen, the Netherlands

**Keywords:** Education, Medical, Podcast, Healthcare professionals, Patients, Communication

## 1 A patient’s experience

*“It started on a Friday morning. I was on my way to work and after cycling for about 500 meters, I started to feel nauseous. It was a nausea so strong that I couldn't pedal any further. So I instinctively knew something was wrong. I called the emergency department, where I was head of the department at the time, and told them I was on my way. Once I arrived, my team got to work as with any other patient. From that moment, I transitioned from being a doctor who is in charge, to a patient who has to blindly step into the rollercoaster and just let it happen.” […] “That period taught me what it truly means to be taken along on the journey of being a patient. When I supervise, I try to make residents understand that what is obvious to you is not necessarily so for the patient. Include them in your considerations and partner with them in decision-making. That is certainly what my experience as a patient has taught me”.*

## 2 Introduction

Above, you hear the voice of a trauma surgeon in one of our episodes, who shares his experience after suffering a myocardial infarction. With this podcast, we offer medical residents a closer look into the dual view of colleagues turned patients. They discuss pivotal moments when they realized they were a patient, the emotional impact, their patient perspectives on care, the influence on their professional approach and the lessons they wish to convey to others.

Patient narratives enhance empathy, deepen understanding of illness, improve communication and promote understanding of patient-centered care ([1], [2], [3]). Spoken narratives, have been shown to be more memorable than written narratives (4). True stories, with personal anecdotes, may provide a more impactful shift in perspective and deepen understanding of the patient experience (5).

Previous research suggests that healthcare-experienced patients might be especially effective as teachers (6,7). They uniquely blend personal illness narratives with clinical expertise, creating valuable learning opportunities in medical education.

Despite its learning potential, structural integration of the patient journey in (postgraduate) medical education remains scarce, while patient-provider interactions predominantly occur during this phase of training (5). The aim of our podcast is to encourage listeners to engage in self-reflection regarding their interactions with patients according their professional behavior. Additionally, we aim to inspire listeners to integrate the lessons learned from colleagues' patient experiences into their own practice.

## 3 Methods

### 3.1 Podcast

We developed five episodes. Healthcare professionals with a patient experience within our medical center were personally approached to determine whether they wanted to participate. We designed the podcast according to Sandar's twelve tips for using podcast effectively in medical education (4). We produced the podcast without visual elements to preserve the vulnerability and intimacy of the stories.

### 3.2 Residents

All medical residents from three different departments (neurology, psychiatry and surgery) were invited to participate. We selected these three departments to maintain disciplinary differences in patient contact time.

### 3.3 Evaluation

The evaluation consists of a survey with 14 questions (Appendix A) about technical aspects of the podcast, the content and what listening has yielded. To complete the evaluation, we interviewed the healthcare professionals about their experience recording the episode.

### 3.4 Data analysis

The results of the survey were collected by MvH and KvD. The interviews were transcribed by an external partner and thematically analysed with atlas.ti 24.0.0.

### 3.5 Ethical approval

This research was presented to the medical ethics review committee of the eastern Netherlands and was stated as not subject to review by an ethics committee. Written informed consent was obtained from the interviewees and the residents.

## 4 Results

Twenty-five medical residents listened to the podcast and filled out the evaluation.

### 4.1 Residents experiences

#### 4.1.1 Reflection on professional behavior

Residents responded that listening to the episodes contributed to increasing awareness of their professional behavior. One resident (R1) said: *‘I learned that we aren't always aware of the non-verbal signs that we (sometimes unconsciously) send’.* Other reactions were: *‘communication, which is clear to the doctor, can simply be interpreted differently by a patient’* (R2) and *‘it is important to really listen to a patient and try to find out what the patient says without pronouncing it’* (R2).

#### 4.1.2 Reflection on empathy

Residents noted a deeper understanding of what it means to be a patient. One of them (R3) mentioned ‘*patients are often in an uncertain and vulnerable position, so it is important to keep to the appointments that have been made’.* Another said: ‘*I can empathize more with patients and therefore show better understanding’* (R4).

#### 4.1.3 Value of switch in identity experienced by colleagues

The patient experience of a fellow healthcare professional has a potential influence on the educational outcomes. As one respondent elucidated: *‘hearing form a colleague about their patient experience proves more memorable than hearing from non-medical patients. While lay patient stories are also interesting, they often lack the insight into the practical challenges inherent in our work. Colleagues have a clear understanding of what is practically feasible for doctors and the significant impact it can have on patients. Therefore they provide practical tips that can easily be applied to your daily work’* (R5)*.*

### 4.2 Interviewees' experiences

#### 4.2.1 Experience of participation

Unanimously, the interviewees experienced their participation as positive. They were all in a different stage of their patient journey, which resulted in different experiences with sharing their stories. One of them experienced the recording as a reflective moment: *‘for me it was really another piece of reflection. Okay, what actually happened and how exactly do I feel about this’* (I1)*.* After editing the episodes, interviewees were given the opportunity to listen to their story. *‘It was such an honest story, I couldn't be more myself’* (I3).

#### 4.2.2 Value on professional approach

A recurring theme was the value of their patient experiences on their professional approach. *‘When I listened back to the episode I realized: I have really started to do that differently since I became a patient. I always ask how information from the last visit came across’* (I1). *‘You are so thrown off balance when you get bad news. Completely different things will then come into play, in every patient. I have come to recognize this better’* (I2), *‘and not only the patients, but also their relatives. For them, it is also intense and they need attention too. The podcast reminds me to really see them’* (I2)*.*

#### 4.2.3 Lessons learned with residents

The healthcare professionals hoped to share their lessons learned with young colleagues. *‘No matter how empathic you are, you only truly see what is important when you are on the other side of the perspective* (I2)*. ‘It is important to create some moments in which you pause and reflect on yourself’* (I3)*.*

## 5 Discussion

Our podcast series with the dual view of colleagues turned patients, was received with great enthusiasm and positive reactions. Residents reported that the podcast contributes to awareness of their professional behavior.

We didn't assess subsequent changes in residents' behavior which may be considered as a study limitation. However, our primary aim was to encourage listeners to engage in self-reflection regarding their interactions with patients and colleagues, making self-reported surveys an appropriate measurement method.

In accordance with previous research, the concept was designed on several success factors for podcasts in medical education. The interview format improves the listening experience and the episodes contain humor and personal anecdotes (5,8). We believe these factors contributed to the positive reactions we received. Narrative use in medical education is not new and supports a transformation in understanding illness (3). At the same time, the use of narrative podcast as educational tools remains uncommon, despite the fact that the medium meets the learning needs in adult learning. While some examples exist in psychiatry, they differ in format from ours (9).

## 6 Innovation

As far as we know, we are the first to incorporate a podcast in continuing medical education to share patient narratives. In oncology, a co-created podcast on cancer survivorship has fostered social support through shared experiences among survivors, caregivers and medical professionals (10). Narratives told by healthcare professionals offer a unique opportunity to broaden students perspectives within the shared challenges of care. This encourages self-reflection as it uniquely blends professional identity formation with one's personal identity.

Compared to other podcast series, this podcast is applicable to all medical training years and specialties. It stimulates interdisciplinary education and builds bridges between more formal theoretical education and informal experiential learning.

## 7 Conclusion

Our podcast stimulates self-reflection on professional behavior within the possibilities of the medical world. The episodes also expand residents' perspective with the patient perspective. The knowledge of healthcare professionals about the working field is of added value in obtaining achievable learning points.

## 8 Future implications

Currently we are expanding our podcast library and we aim to further integrate this initiative into medical education, promoting a sustainable model for learning from patient experiences.

## CRediT authorship contribution statement

**M.A. van Helvoort:** Writing – original draft, Resources, Project administration, Methodology, Investigation, Formal analysis, Data curation, Conceptualization. **K.J.A. van Dijsseldonk:** Writing – original draft, Methodology, Investigation, Data curation, Conceptualization. **B. Post:** Supervision, Project administration, Methodology, Investigation, Conceptualization.

## Declaration of competing interest

The authors declare that they have no known competing financial interests or personal relationships that could have appeared to influence the work reported in this paper.

## Appendix A Supplementary data

Appendix A: survey for evaluation Image 1
