# Supplementary material for: When physicians become patients: A podcast series for learning from patient experiences
Source: PEC Innov. 2025 Aug 22;7:100423. doi: 10.1016/j.pecinn.2025.100423 (PMC12409975; doi:10.1016/j.pecinn.2025.100423)
Supplement: Supplementary file 1 — Appendix A: survey for evaluation [file mmc1.docx]

**Appendix A: Survey**

1. The availability of the podcast was:
   1. Good
   2. Moderate
   3. Bad
2. On which device did you listen to the episodes?
3. I listened to the podcast while doing something else, namely..
4. The length of the episodes was:
   1. Far too long
   2. Too lang
   3. Just right
   4. Too short
   5. Far too short
5. The technical quality of the podcast was:
   1. Good
   2. Moderate
   3. Bad
6. The content of the episodes was:
   1. Monotonous
   2. Neutral
   3. Varied
7. This episode was the **most** contributing, because
8. This episode was the **less** contributing, because
9. I am curious about new episodes:
   1. Not at all
   2. No
   3. Neutral
   4. Yes
   5. Very
10. I would recommend the podcast to my colleagues
    1. Not at all
    2. No
    3. Neutral
    4. Yes
    5. Very
11. How can we improve the podcast?
12. This podcast makes me reflect on my own communication
    1. Yes
    2. No
13. What did you learn from the podcast?
14. What is changed in your daily work?
